# Distinct brain pathologies associated with Alzheimer’s disease biomarker-related phospho-tau 181 and phospho-tau 217 in *App* knock-in mouse models of amyloid-β amyloidosis

**DOI:** 10.1093/braincomms/fcac286

**Published:** 2022-11-06

**Authors:** Yu Hirota, Yasufumi Sakakibara, Kyoko Ibaraki, Kimi Takei, Koichi M Iijima, Michiko Sekiya

**Affiliations:** Department of Neurogenetics, Center for Development of Advanced Medicine for Dementia, National Center for Geriatrics and Gerontology, Obu, Aichi 474-8511, Japan; Research Fellow of Japan Society for the Promotion of Science, Chiyoda-ku, Tokyo 102-0083, Japan; Department of Neurogenetics, Center for Development of Advanced Medicine for Dementia, National Center for Geriatrics and Gerontology, Obu, Aichi 474-8511, Japan; Department of Neurogenetics, Center for Development of Advanced Medicine for Dementia, National Center for Geriatrics and Gerontology, Obu, Aichi 474-8511, Japan; Department of Neurogenetics, Center for Development of Advanced Medicine for Dementia, National Center for Geriatrics and Gerontology, Obu, Aichi 474-8511, Japan; Department of Neurogenetics, Center for Development of Advanced Medicine for Dementia, National Center for Geriatrics and Gerontology, Obu, Aichi 474-8511, Japan; Department of Experimental Gerontology, Graduate School of Pharmaceutical Sciences, Nagoya City University, Nagoya 467-8603, Japan; Department of Neurogenetics, Center for Development of Advanced Medicine for Dementia, National Center for Geriatrics and Gerontology, Obu, Aichi 474-8511, Japan; Department of Experimental Gerontology, Graduate School of Pharmaceutical Sciences, Nagoya City University, Nagoya 467-8603, Japan

**Keywords:** Alzheimer’s disease, biomarker, phosphorylated-tau, amyloid-β, *App* knock-in mouse

## Abstract

Phospho-tau 217, phospho-tau 231 and phospho-tau 181 in cerebrospinal fluid and plasma are promising biomarkers for the diagnosis of Alzheimer’s disease. All these p-tau proteins are detected in neurofibrillary tangles in brains obtained post-mortem from Alzheimer’s disease patients. However, increases in p-tau levels in cerebrospinal fluid and plasma during the preclinical stage of Alzheimer’s disease correlate with amyloid-β burden and precede neurofibrillary tangles in brains, suggesting that these p-tau proteins are indicative of amyloid-β-mediated brain pathology. In addition, phospho-tau 217 has greater sensitivity than phospho-tau 181, though it is unclear whether each of these p-tau variants contributes to the same or a different type of neuropathology prior to neurofibrillary tangle formation. In this study, we evaluated the intracerebral localization of p-tau in *App* knock-in mice with amyloid-β plaques without neurofibrillary tangle pathology (*App^NLGF^*), in *App* knock-in mice with increased amyloid-β levels without amyloid-β plaques (*App^NL^*) and in wild-type mice. Immunohistochemical analysis showed that phospho-tau 217 and phospho-tau 231 were detected only in *App^NLGF^* mice as punctate structures around amyloid-β plaques, overlapping with the tau pathology marker, AT8 epitope phospho-tau 202/205/208. Moreover, phospho-tau 217 and phospho-tau 202/205/208 colocalized with the postsynaptic marker PSD95 and with a major tau kinase active, GSK3β. In contrast and similar to total tau, phospho-tau 181 signals were readily detectable as fibre structures in wild-type and *App^NL^* mice and colocalized with an axonal marker neurofilament light chain. In *App^NLGF^* mice, these phospho-tau 181-positive structures were disrupted around amyloid-β plaques and only partially overlapped with phospho-tau 217. These results indicate that phospho-tau 217, phospho-tau 231 and a part of phospho-tau 181 signals are markers of postsynaptic pathology around amyloid-β plaques, with phospho-tau 181 also being a marker of axonal abnormality caused by amyloid-β burden in brains.

## Teaser

Hirota *et al.* report that phospho-tau 217, phospho-tau 231 and phospho-tau 181, cerebrospinal fluid and plasma biomarkers for preclinical Alzheimer’s disease, are associated with postsynaptic pathology around amyloid-β plaques, while phospho-tau 181 also represents axonal abnormality in the absence of neurofibrillary tangle pathology in *App* knock-in mouse models of amyloid-β amyloidosis.

## Graphical Abstract

**Graphical abstract:**
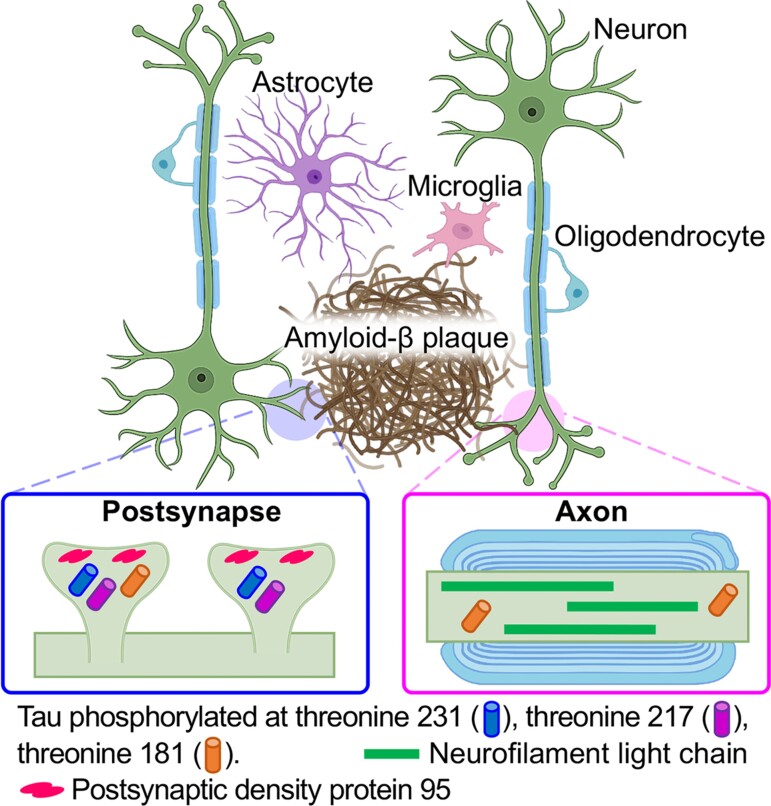

## Introduction

Alzheimer’s disease is a progressive neurodegenerative disease and a major cause of senile dementia worldwide.^1^ Pathologically, Alzheimer’s disease is characterized by senile plaques composed of amyloid-β peptides (Aβ) and neurofibrillary tangles (NFTs) composed of hyperphosphorylated microtubule-associated tau proteins, resulting in brain atrophy.^2^ Investigations of the mechanisms underlying Alzheimer’s disease pathogenesis have indicated that the accumulation of Aβ in the brain induces chronic neuroinflammation, tau pathology and irreversible neuron loss.^3–5^ Methods to definitively diagnose the preclinical stage of Alzheimer’s disease are needed to develop disease-modifying treatments.

Positron emission tomography (PET) and the detection of biomarkers such as Aβ and tau in cerebrospinal fluid (CSF) and plasma are highly accurate methods of detecting brain pathology in patients with Alzheimer’s disease.^6^ Amyloid PET has revealed that Aβ accumulation in brains can be detected more than two decades before the clinical onset of Alzheimer’s disease.^7–10^ The level of Aβ42, a major constituent of Aβ plaques, in CSF was found to correlate negatively with Aβ burden.^11^ The Aβ40-to-Aβ42 ratio in plasma, as determined by immunoprecipitation–mass spectrometry-based methods, is a minimally invasive, cost-effective and highly sensitive blood-based biomarker for Alzheimer’s disease.^12,13^ In contrast, tau-PET has shown that tau-positivity coincides with the clinical onset of Alzheimer’s disease and that affected brain areas correlate with clinical manifestations.^14^ The levels of total tau and tau phosphorylated at Thr181 (p-tau 181) in CSF, in combination with Aβ, are additional diagnostic biomarkers for Alzheimer’s disease.^15–17^ Moreover, increases in the levels of neurofilament light chain (NfL), a general biomarker for neurodegeneration,^18^ in CSF and plasma are predictive of cognitive impairment and neurodegeneration in Alzheimer’s disease.^19^

In addition to p-tau 181, tau phosphorylated at Thr217 (p-tau 217) and Thr231 (p-tau 231) in CSF and plasma is diagnostic of Alzheimer’s disease during the preclinical period with high accuracy.^16,20–24^ Although all of these p-tau species are present in pretangles and NFTs in post-mortem Alzheimer’s disease brains,^25–27^ p-tau 181 and p-tau 217 levels in CSF begin to rise two decades before tau-PET positivity in patients showing dominant inheritance of Alzheimer’s disease.^28^ The levels of p-tau 181 and p-tau 217 start to increase slightly before Aβ-PET positivity and correlate with Aβ pathology in cognitively unimpaired individuals.^29,30^ Interestingly, p-tau 217 detects Alzheimer’s disease slightly earlier than p-tau 181 during the preclinical period,^24,28^ whereas plasma p-tau 181 levels gradually increase from the preclinical stage of Alzheimer’s disease to mild cognitive impairment and dementia.^16,31^ Taken together, these findings indicate that increased levels of p-tau 181, p-tau 217 and p-tau 231 are promising biomarkers for the detection of the preclinical stage of Alzheimer’s disease as being correlated with Aβ pathology.^32^ However, the mechanism underlying the correlations between these p-tau species and Aβ pathology and whether these p-tau species are characteristic of the same or different brain pathologies before definitive NFT formation remain unclear.

The relationships of p-tau 181, p-tau 217 and p-tau 231 signals with neuropathology induced by Aβ accumulation in brains were investigated using *App* knock-in mouse models. *App^NLGF^* mice harbour three familial Alzheimer’s disease mutations, Swedish (NL), Arctic (G) and Beyreuther/Iberian (F) and exhibit cognitive deficits and neuroinflammation accompanied by progressive Aβ pathology in the brain parenchyma, whereas *App^NL^* mice carry only the Swedish (NL) mutation and overproduce human Aβ without the formation of Aβ plaques for up to 24 months.^33,34^ Neither *App^NLGF^* nor *App^NL^* mice develop NFTs or neuropil threads composed of tau aggregates, suggesting that these mouse models recapitulate the preclinical stage of Alzheimer’s disease. Immunohistochemical analysis using p-tau-specific antibodies in combination with a series of neuronal and glial marker antibodies revealed that p-tau 217, p-tau 231 and some p-tau 181 signals were associated with postsynaptic pathology around Aβ plaques, whereas p-tau 181 also reflects axonal abnormalities due to Aβ burden. These results suggested that the biomarkers p-tau 217 and p-tau 181 represent distinct types of brain pathology induced by Aβ plaques before pretangle or NFT formation.

## Materials and methods

### Animals

Experiments were performed using 6- or 24-month-old male C57BL/6J and *App* knock-in (*App^NL^*, *App^NLGF^*) mice and 24-month-old female C57BL/6J and *App^NLGF^* mice. *App* knock-in mice on a C57BL/6J genetic background^33^ were obtained from the RIKEN Center for Brain Science (Wako, Japan) and maintained at the Institute for Animal Experimentation, the National Center for Geriatrics and Gerontology, as described previously.^35^ After weaning, all mice were housed socially in same-sex groups (3–5 animals per cage) in a controlled environment (constant temperature 22 ± 1°C, humidity 50–60%) under a 12 h light/dark cycle (lights on at 7:00; lights off at 19:00), with access to food and water *ad libitum*. All handling and experimental procedures were performed in accordance with the NIH Guide for the Care and Use of Laboratory Animals and other national regulations and policies with the approval of the Animal Care and Use Committee at the National Center for Geriatrics and Gerontology, Japan (approval number: 2–45).

### Tissue preparation

All animals were deeply anaesthetized by intraperitoneal administration of a combination of medetomidine (0.3 mg/kg), midazolam (4.0 mg/kg) and butorphanol (5.0 mg/kg), and immediately perfused intracardially with ice-cold saline, followed by 4% paraformaldehyde in 0.1 M phosphate buffer , as previously described.^35^ Whole brains were collected and immersed in the same fixative at 4°C overnight. For cryoprotection, the fixed brains were transferred to 20% and then 30% sucrose in 0.1 M phosphate buffer at 4°C until the tissues sank. After freezing rapidly in cold isopentane, the brains were stored at −80°C until use. Brains were sectioned coronally at 25 μm with a cryostat (CM3050S; Leica Biosystems, Germany). Tissue sections were stored in cryoprotectant [30% glycerol and 30% ethylene glycol in phosphate-buffered saline (PBS)] at −20°C until immunostaining.

### Immunohistochemistry

Immunohistochemical staining was performed as described previously.^35^ Briefly, tissue sections were washed with PBS containing 0.1% Triton X-100 (PBS-T) and blocked in a buffer containing 5% normal goat serum, 0.5% bovine serum albumin (BSA), and 0.3% Triton X-100 in PBS for 1 h. The sections were then incubated overnight at 4°C with primary antibodies (Supplementary Table 1) diluted in 3% normal goat serum, 0.5% BSA and 0.3% Triton X-100 in PBS. For anti-Aβ immunostaining, antigen retrieval was performed by incubating the sections for 5 min in 70% formic acid before blocking. The tissues were then washed three times with PBS-T and incubated for 2 h with appropriate fluorescent secondary antibodies (Supplementary Table 1) in the same dilution buffer. The sections were again washed three times with PBS-T and incubated with 4',6-Diamidino-2-phenylindole (DAPI) (2 μg/mL in PBS) for 5 min. For the detection of Aβ amyloidosis, the tissues were stained with 1-fluoro-2,5-bis (3-carboxy-4-hydroxystyryl) benzene (FSB) solution (10 μg/mL in 50% ethanol) for 30 min. The sections were mounted in Aqua-Poly/Mount (Polysciences Inc., USA) and stored at 4°C until image acquisition.

### Image acquisition and analysis

Images were acquired using either an LSM700 or an LSM780 confocal laser-scanning microscope (Carl Zeiss, Germany) fitted with 20×, 40× or 63× objectives. Images were acquired from the cortex, the CA1 region of the hippocampus and the locus coeruleus (LC). Laser and detection settings were maintained for each immunostaining. All image processing and analysis were performed with Fiji software. Z-stack confocal images were reconstructed with a maximum intensity projection. For high-magnification images, as indicated by the dashed orange squares in the figures, orthogonal views of confocal z-stacks were reconstructed using a Fiji plugin . In the orthogonal views of the white dashed line in the high-magnification images, the *x*- and *y*-axes were aligned to focus on the p-tau 231 or AT8 signal. For three-dimensional (3D) reconstructions, images were processed using a plugin (volume viewer) in max projection mode. In the cross-sectional view of the green line in the reconstructed 3D image, the *x*- and *z*-axes were aligned to focus on the AT8 signal using the slice and borders mode in the volume viewer plugin.

### Quantification of the ratio of p-tau-positive Aβ plaques and the number of p-tau signals around Aβ plaques

To quantify the number of Aβ plaques associated with p-tau signals in the cortical regions, we captured images using an LSM 700 confocal microscope with a 20× objective. The number of total Aβ plaques and that of p-tau-positive Aβ plaques were manually counted from acquired images by an unbiased individual. The values were expressed as the ratio of the number of p-tau-positive Aβ plaques to the number of total Aβ plaques. The number of p-tau-positive signals around Aβ plaques was manually counted from acquired images by an unbiased individual. The values were expressed as the number of p-tau signals per Aβ plaque.

### Quantification of the rate of colocalization of p-tau signals

To quantify the rate of colocalization of p-tau punctate signals in the cortical regions, we captured images using an LSM 700 confocal microscope with a 40× objective. Each p-tau signal was manually counted from acquired images by an unbiased individual and the rate of colocalization was calculated between p-tau 217 and p-tau 202/205/208 (AT8), p-tau 231 and p-tau 202/205 (AH36), p-tau 217 and p-tau181, p-tau 217 and PSD95, p-tau 231 and PSD95 or p-tau 181 and PSD95.

### Statistical analysis

The data are presented as the mean ± standard error of the mean. Unpaired Student’s *t*-test (GraphPad Prism 9, GraphPad software) was used to determine statistical significance (*P < 0.05, **P < 0.01 and ***P < 0.001).

### Data availability

All the data generated or analysed during this study are included in this published article.

## Results

### Detection of p-tau 217, p-tau 231 and p-tau 202/205/208 (AT8) signals as punctate structures around Aβ plaques in the brains of *App^NLGF^* mice

Since the appearance of p-tau 181, p-tau 217 and p-tau 231 in the CSF and blood correlates with Aβ pathology but not with NFT pathology, we utilized *App* knock-in mice with massive Aβ plaque in the brain parenchyma without NFTs (*App^NLGF^* mice) to investigate the relationships between the locations of these p-tau signals and Aβ-induced neuropathology. We chose to use *App* knock-in mice rather than *App* transgenic mice because transgenic overexpression of *App* by itself causes axonal transport defects^36–39^ and may affect the distribution and metabolism of axonal proteins, including tau. As age-matched controls, we utilized *App* knock-in mice with increased soluble Aβ levels but no Aβ plaques (*App^NL^* mice) and their genetic background C57BL/6J wild-type (WT) mice without expression of human Aβ.

**Figure 1:**
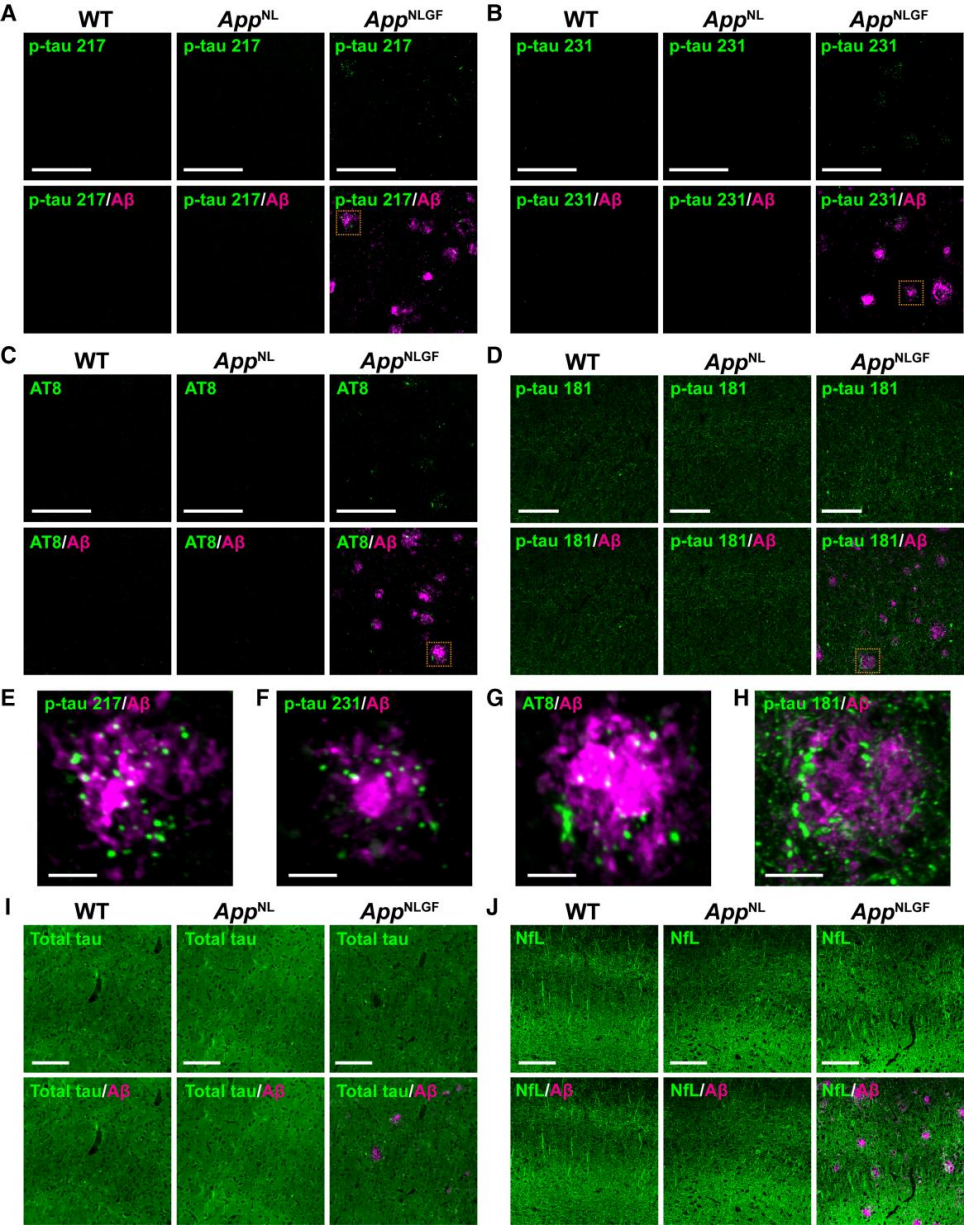
**P-tau 217, 231, 202/205/208 (AT8) and 181 are present around Aβ plaques in *App^NLGF^* mice.** Representative images of cortices from frozen coronal brain sections immunostained with antibodies against p-tau 217 (**A**, **E**), p-tau 231 (**B**, **F**), p-tau 202/205/208 (AT8) (**C**, **G**), p-tau 181 (**D**, **H**), total tau (**I**) and NfL (**J**) in combination with an anti-Aβ antibody. Higher magnification views of the dashed orange squares in **A**–**D** are shown in **E**–**H**, respectively. Scale bars, 100 μm in **A**–**D, I**–**J** and 10 μm in **E**–**H**.

The brain sections from 6- and 24-month-old male *App^NLGF^*, *App^NL^* and WT mice were stained with antibodies against p-tau 217 and p-tau 231, which are CSF and plasma biomarkers of the preclinical stage of Alzheimer’s disease.^21,23,24^ These antibodies detected p-tau 217 and p-tau 231 signals in the cortex (Fig. 1A and B) and hippocampus (Supplementary Fig. 1A and B) of *App^NLGF^* mice, while no clear signal was detected in the brains of either *App^NL^* or WT mice (Fig. 1A and B). The signals in *App^NLGF^* mice appeared as punctate structures closely associated with Aβ plaques (Fig. 1E and F). The AT8 epitope p-tau 202/205/208^40^ is a marker for NFT and neuritic pathology in the brains of patients with Alzheimer’s disease and mouse models of Alzheimer’s disease.^27,41^ Like p-tau 217 and p-tau 231, p-tau 202/205/208 (AT8) signals were only detected in the brains of *App^NLGF^* mice as punctate structures around Aβ plaques (Fig. 1C and G). Similar staining patterns of p-tau 217, p-tau 231 and p-tau 202/205/208 (AT8) were observed in 24-month-old female *App^NLGF^* mice (Supplementary Fig. 2A, B and C).

To ask whether these p-tau signals were increased along with the exacerbation of Aβ pathology, we quantified the ratio of the number of p-tau 217- or p-tau 231-positive Aβ plaques to that of the total number of Aβ plaques in 6- and 24-month-old *App^NLGF^* mice. Compared with 6-month-old mice, the ratio of p-tau-positive Aβ plaques was significantly increased in 24-month-old *App^NLGF^* mice (Supplementary Fig. 3A, B and D). In addition, the number of p-tau 217 or p-tau 231 signals around Aβ plaque was significantly increased in 24-month-old *App^NLGF^* mice than in 6-month-old *App^NLGF^* mice (Supplementary Fig. 3A, B and E). Taken together, these results indicate that p-tau 217, p-tau 231 and p-tau 202/205/208 (AT8) signals are present in similar punctate structures and are closely associated with Aβ plaques in mouse brains.

### Detection of p-tau 181 signals as fibre structures in the brains of WT and *App^NL^* mice and disruption of these structures around Aβ plaques in *App^NLGF^* mice

The concentration of p-tau 181 in CSF has long been used in clinical practice as a biomarker for Alzheimer’s disease. Increases in plasma p-tau 181 have also been found to differentiate Alzheimer’s disease from non-Alzheimer’s disease and predict cognitive decline during the preclinical and prodromal stages of Alzheimer’s disease.^16,31,42,43^ Interestingly, p-tau 217 concentrations in CSF and plasma have been found to be more sensitive than p-tau 181 in detecting Alzheimer’s disease during preclinical stages.^20,44,45^ To determine whether the localizations of p-tau 181 and p-tau 217 are similar, brain sections from 6- and 24-month-old *App^NLGF^*, *App^NL^* and WT mice were stained with an antibody against p-tau 181. In contrast to p-tau 217, p-tau 231 and p-tau 202/205/208 (AT8) (Fig. 1A–C and Supplementary Fig. 1A–C), p-tau 181 signals were detected as fibre structures of the cortex and hippocampus in all three mouse genotypes (Fig. 1D and Supplementary Fig. 1D). However, p-tau 181 signals were present as aberrant fibre structures around Aβ plaques in *App^NLGF^* mice (Fig. 1D and H). Similar staining patterns of p-tau 181 were observed in 24-month-old female *App^NLGF^* mice (Supplementary Fig. 2D).

To ask whether these p-tau 181-positive aberrant fibre structures were increased along with the exacerbation of Aβ pathology, we quantified the ratio of the number of Aβ plaques associated with p-tau 181-positive aberrant fibre structures to that of the total number of Aβ plaques in 6- and 24-month-old *App^NLGF^* mice. Compared with 6-month-old mice, the ratio of p-tau-positive Aβ plaques was significantly increased in 24-month-old *App^NLGF^* mice (Supplementary Fig. 3C and F). These distribution patterns were similar to those of total tau, although total tau was much more abundant and ubiquitous than p-tau 181 (Fig. 1I and Supplementary Fig. 1E). To determine whether prominent axonal degeneration occurs in *App^NLGF^* mice, brain sections were stained with an antibody against NfL, a biomarker of neurodegeneration in many neurodegenerative conditions.^18,19,46,47^ Similar to total tau, NfL was abundant and ubiquitous in all three mouse genotypes, including *App^NLGF^* mice, although the NfL signal was disrupted in areas of Aβ plaques (Fig. 1J and Supplementary Fig. 1F). Taken together, these results indicate that the distribution pattern of p-tau 181 differs from that of p-tau 217-, p-tau 231- or p-tau 202/205/208 (AT8) in the brains of *App^NLGF^* mice.

### Colocalization of p-tau 217, p-tau 231 and 202/205/208 (AT8), but not p-tau 181, with amyloid plaques in *App^NLGF^* mice

**Figure 2:**
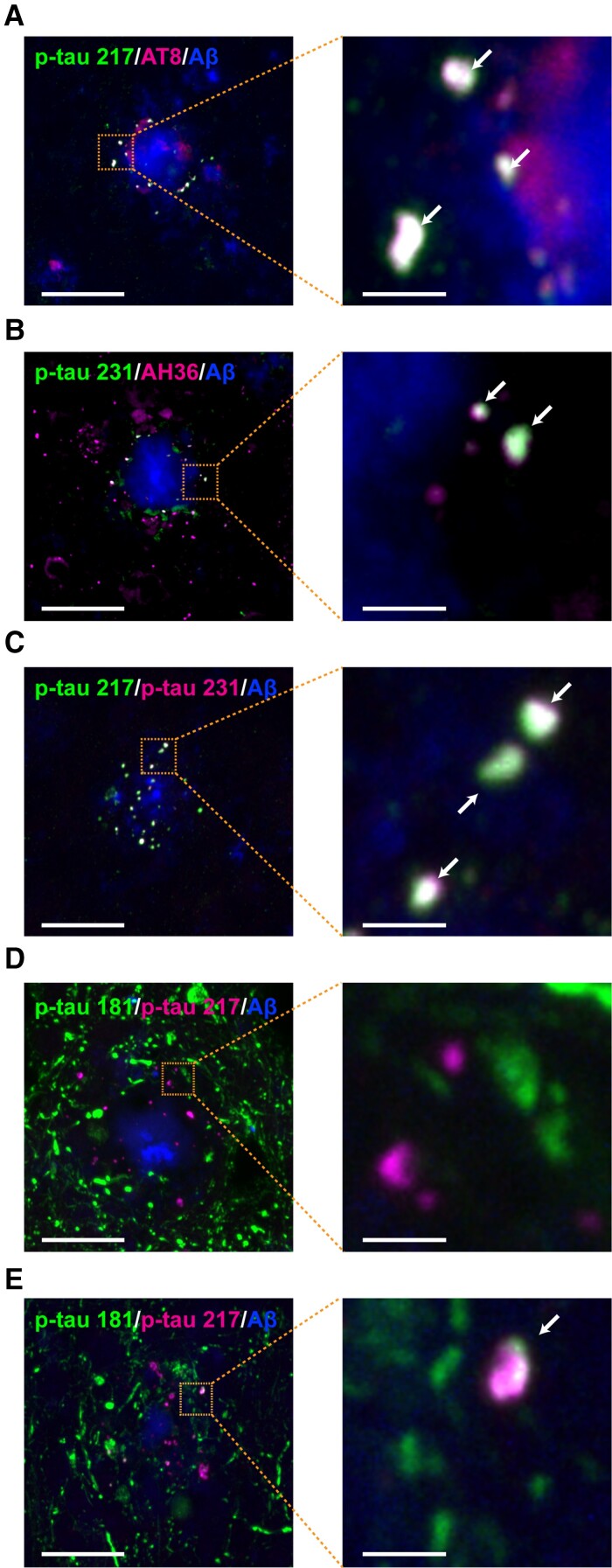
**P-tau 217, 231, 202/205/208 (AT8) and 202/205 (AH36) colocalize, but p-tau 181 does not.** Representative images of cortices from frozen coronal brain sections immunostained with antibodies against (**A**) p-tau 217 and p-tau 202/205/208 (AT8), (**B**) p-tau 231 and p-tau 202/205 (AH36), (**C**) p-tau 217 and p-tau 231 and (**D**, **E**) p-tau 181 and p-tau 217. Aβ plaques are detected by staining with FSB. Scale bars, 20 µm. Higher magnification views of the dashed orange squares are shown on the right side. White arrows show colocalization. Scale bars, 2.5 μm.

Because the punctate structures of p-tau 217, p-tau 231 and p-tau 202/205/208 (AT8) were similar (Fig. 1A–C and 1E–G), brain sections from 24-month-old *App^NLGF^* mice were stained with antibodies against p-tau 217 and p-tau 202/205/208 (AT8), against p-tau 231 and p-tau 202/205 (AH36) or against p-tau 217 and p-tau 231. As expected, each of these pairs of signals colocalized around Aβ plaques (Fig. 2A–C). Quantification of the rate of colocalization of p-tau 217 and p-tau 202/205/208 (AT8) around four amyloid plaques showed that an average of 82.0% of punctate signals positive for p-tau 217 colocalized with those of p-tau 202/205/208 (AT8). Similarly, an average of 91.6% of punctate signals positive for p-tau 217 colocalized with those of p-tau 231. In contrast, co-staining of brain sections from 24-month-old *App^NLGF^* mice with antibodies against p-tau 181 and p-tau 217 showed that the majority of p-tau 181 signals did not colocalize with those of p-tau 217 (Fig. 2D), although some did (Fig. 2E). Quantification of the rate of colocalization of p-tau 181 and p-tau 217 around four amyloid plaques showed that an average of 12.5% of punctate signals positive for p-tau 181 colocalized with those for p-tau 217. Taken together, these results suggest that signals for both p-tau 181 and p-tau 217 are altered around Aβ plaques, although they may reflect different aspects of neuronal pathology induced by Aβ burden in the mouse brain.

### Absence of tau pathology positive for p-tau 217, p-tau 231 and p-tau 181 in the LC of *App^NLGF^* mouse brains

The LC noradrenergic neurons in the brainstem are among the earliest brain regions to develop NFT pathology in patients with Alzheimer’s disease. To determine whether p-tau 217 or p-tau 231 was present in the LC of aged mouse brains, brain sections containing LC regions from 24-month-old *App^NLGF^*, *App^NL^* and WT mice were stained with antibodies against p-tau 217 or p-tau 231, along with antibodies against tyrosine hydroxylase, a marker for noradrenergic neurons. Neither p-tau 217 nor p-tau 231 was detected in the LC of all genotypes, suggesting that either ageing or Aβ pathology in the cortex was not sufficient to induce tau phosphorylation at these sites in the LC (Supplementary Fig. 4A and B). To determine whether p-tau 181 and total tau were accumulated and formed tau pathology in the noradrenergic neurons of the LC, brain sections of aged *App^NLGF^*, *App^NL^* and WT mice were stained with antibodies against p-tau 181 and total tau, but there were no differences in their staining patterns in any of these mice (Supplementary Fig. 4C and D). These results confirmed that neither ageing nor Aβ pathology in the cortex is sufficient to induce p-tau 217-, p-tau 231- or p-tau 181-positive tau pathology in the LC of the *App^NLGF^* mouse brain.

### Colocalization of p-tau 217, p-tau 231 and p-tau 202/205/208 (AT8) with PSD95, a marker for postsynaptic density of glutamatergic neurons, in the brains of *App^NLGF^* mice

**Figure 3:**
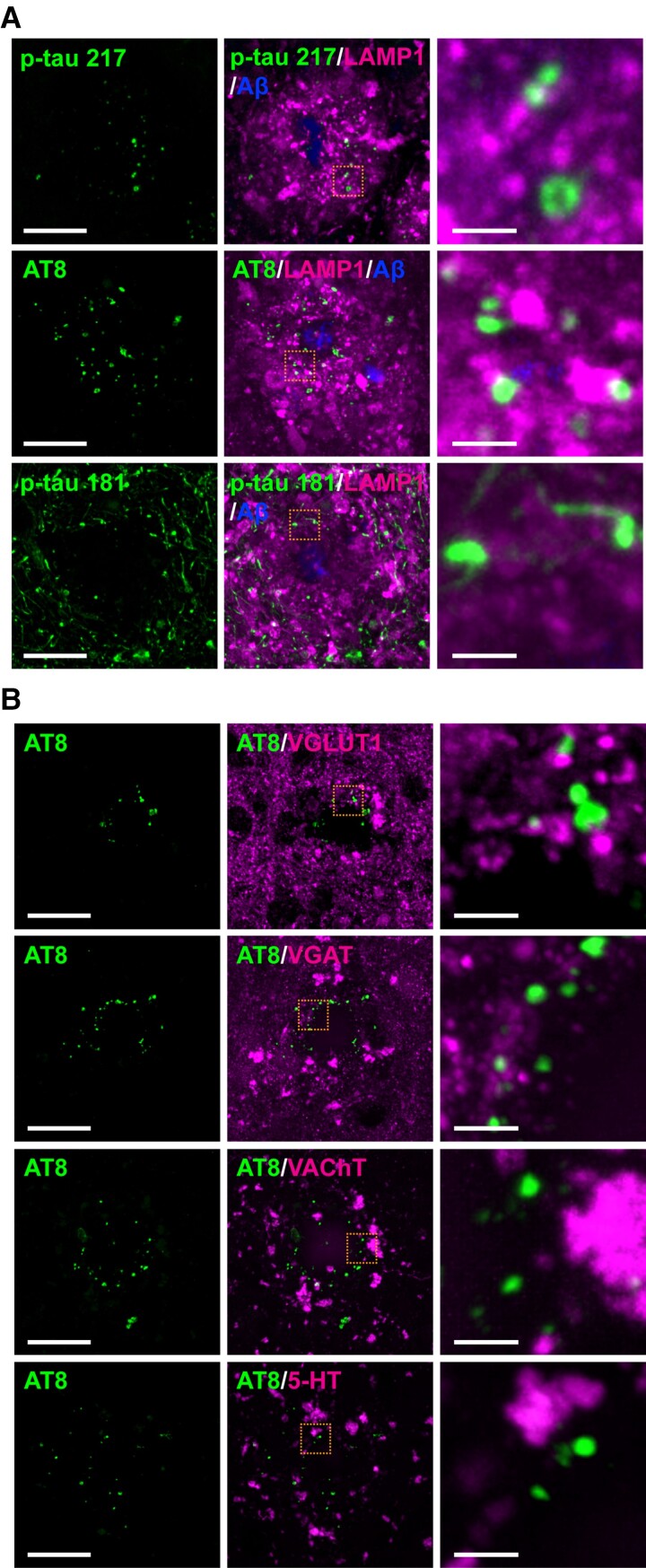
**P-tau 202/205/208 (AT8) is not present at dystrophic neurites or presynaptic terminals.** Representative images of cortices from frozen coronal brain sections immunostained with (**A**) antibodies against p-tau (217, 202/205/208:AT8 or 181) and LAMP1, a marker of dystrophic neurites. Aβ plaques are stained with FSB. (**B**) antibodies against p-tau AT8 and the glutamatergic presynaptic marker VGLUT1, the GABAergic presynaptic marker VGAT, the cholinergic terminal marker VAChT or the serotonergic terminal marker 5-HT. Scale bars, 20 μm in each left panel. Each right panel shows a higher magnification view of the corresponding dashed orange squares; scale bars, 2.5 μm.

To determine the subcellular localization of punctate structures positive for p-tau 217 and p-tau 202/205/208 (AT8) in neurons, we first examined whether these signals colocalized with dystrophic neurites around Aβ plaques. Co-staining of brain sections from 24-month-old *App^NLGF^* mice with antibodies against p-tau 217 or p-tau 202/205/208 (AT8) and lysosomal-associated membrane protein 1 (LAMP1), a marker for dystrophic neurites, showed that neither p-tau 217 nor p-tau 202/205/208 (AT8) signals overlapped with those of LAMP1 (Fig. 3A). In addition, p-tau 181 did not colocalize with LAMP1 (Fig. 3A). These results are consistent with a recent report showing that LAMP1 signals are more abundant than p-tau 202/205/208 (AT8) signals and that they colocalize weakly around Aβ plaques in the hippocampus of the human brain.^48^

**Figure 4:**
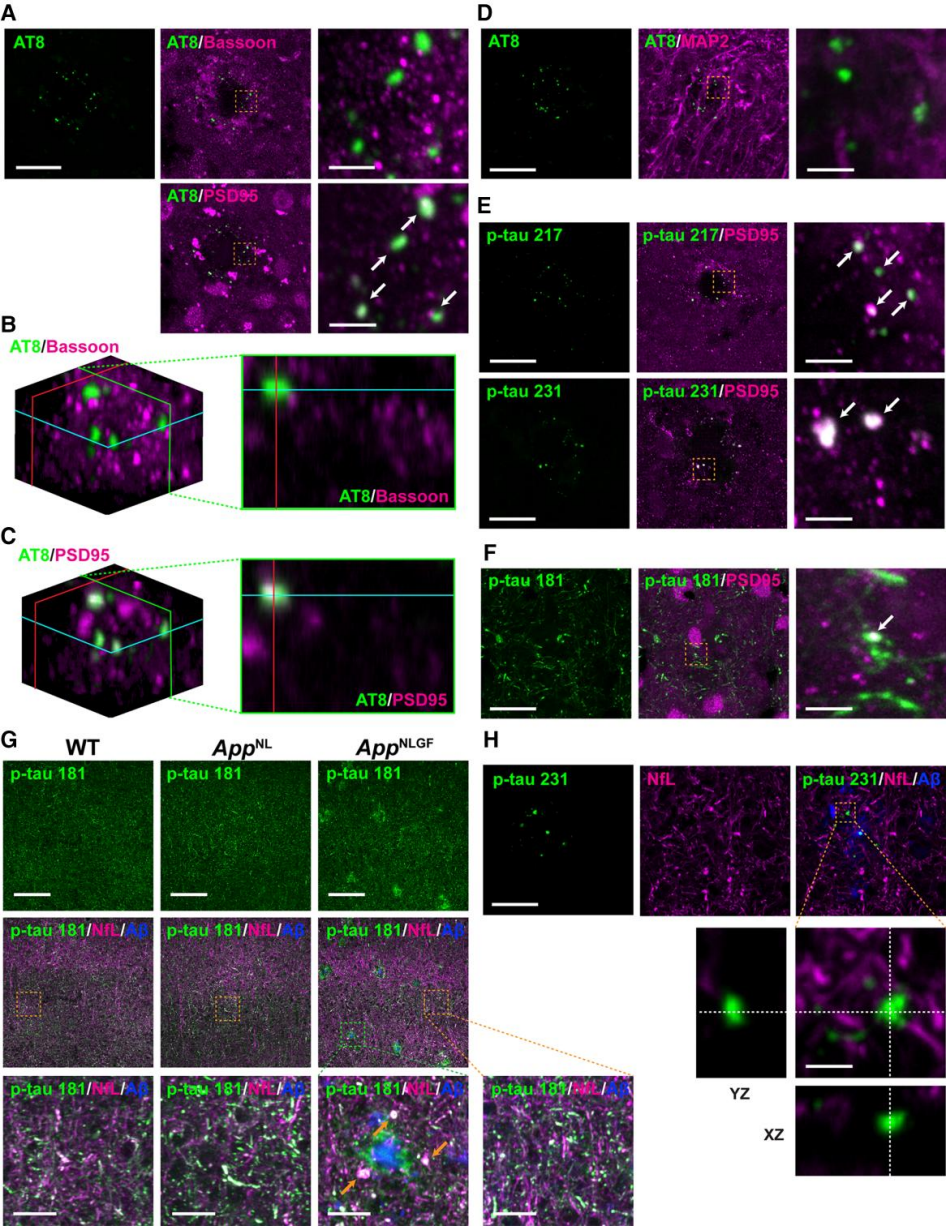
**Colocalization of p-tau 217, 231 and 202/205/208 (AT8) with a postsynaptic marker, PSD95.** (**A**) Representative images of cortices from frozen coronal brain sections immunostained with antibodies against p-tau 202/205/208 (AT8) and the pan-presynaptic marker Bassoon or the glutamatergic postsynaptic marker PSD95. (**B**, **C**) 3D images reconstructed using the Fiji volume viewer plugin, showing that AT8 colocalized with PSD95, but not with Bassoon. A cross-sectional view along the green line in the 3D images is shown. (**D**–**F**) Co-immunostaining of cortices with antibodies against (**D**) AT8 and the neuronal dendritic marker MAP2, (**E-F**) p-tau 217, 231 or 181 and PSD95. Scale bars, 20 μm. The right panel shows higher magnification views of the corresponding dashed orange squares; scale bars, 2.5 μm. White arrows show colocalization. (**G**) Representative images of cortices immunostained with antibodies against p-tau 181 and the axonal marker NfL. Aβ plaques are stained with FSB. Scale bars, 100 μm. The bottom panel shows higher magnification views of the corresponding dashed orange and green squares; scale bars, 10 μm. Orange arrows show aberrant fibre structures. (**H**) Representative images of cortices immunostained with antibodies against p-tau 231 and the axonal marker NfL. Aβ plaques are stained with FSB. Scale bars, 20 μm. Orthogonal views of different planes (*yz*, *xz*) of higher magnification images from the corresponding dashed orange squares are shown on the bottom. Scale bars in the orthogonal views, 2.5 µm.

Tau is a microtubule-binding protein that normally localizes to axons.^49,50^ To determine whether p-tau 217- and p-tau 202/205/208 (AT8)-positive punctate signals localize to axons or presynaptic terminals, brain sections from 24-month-old *App^NLGF^* mice were stained with antibodies against p-tau 202/205/208 (AT8) and various neurotransmitter or presynaptic markers, including the glutamatergic neuronal vesicle marker vesicular glutamate transporter 1 (VGLUT1), the gamma amino butyric acid (GABA)-ergic neuronal vesicle marker vesicular GABA transporter (VGAT), the cholinergic neuronal vesicle marker vesicular acetylcholine transporter(VAChT) and the serotonergic neuronal marker 5-hydroxytryptamine (5-HT) (Fig. 3B), as well as with the Bassoon, a marker for presynaptic cytomatrix protein (Fig. 4A). None of these marker proteins colocalized with p-tau 202/205/208 (AT8), although a cross-sectional view from 3D reconstruction revealed that p-tau 202/205/208 (AT8) signals were often adjacent to those of Bassoon, a marker for presynaptic terminals (Fig. 4B).

Under pathological conditions, tau proteins can erroneously localize to or be locally translated in the dendrites and postsynapses of the brains of patients with Alzheimer’s disease and mouse models of Alzheimer’s disease.^51–55^ Brain sections from 24-month-old *App^NLGF^* mice were therefore stained with antibodies against p-tau 202/205/208 (AT8) and microtubule-associated protein 2 (MAP2), a marker for cell bodies and dendrites of mature neurons, or postsynaptic density protein 95 (PSD95), a marker for postsynaptic density. Although the signals of p-tau 202/205/208 (AT8) and MAP2 did not clearly overlap (Fig. 4D), p-tau 202/205/208 (AT8) and PSD95 signals showed colocalization (Fig. 4A). A cross-sectional view of a 3D reconstruction confirmed that p-tau 202/205/208 (AT8) and PSD95 signals overlapped (Fig. 4C) and were often adjacent to MAP2 signals (Fig. 4D). To further confirm this result, brain sections from 24-month-old *App^NLGF^* mice were co-stained with antibodies against p-tau 217 or p-tau 231 and PSD95. Quantification of the rate of colocalization of p-tau signals and PSD95 around three amyloid plaques revealed that an average of 81.0% of p-tau 217-, 83.3% of p-tau 231- and 83.5% of p-tau 202/205/208 (AT8)-positive punctate signals colocalized with PSD95 (Fig. 4A and E). In contrast, most p-tau 181-positive punctate signals did not colocalize with PSD95, although an average of 7.7% of p-tau 181 signals did (Fig. 4F).

Next, to determine the subcellular localization of p-tau 181-positive structures in neurons, brain sections from 24-month-old *App^NLGF^*, *App^NL^* and WT mice were co-stained with antibodies against p-tau 181 and an axonal marker NfL. The p-tau 181 signals were well colocalized with NfL signals in all genotypes (Fig. 4G, inset with orange squares), and these structures were disrupted around Aβ plaques in *App^NLGF^* mice (Fig. 4G, inset with green square). In contrast, p-tau 231-positive punctate signals were not colocalized with NfL, as expected (Fig. 4H). Taken together, these results suggested that p-tau 217, p-tau 231 and a fraction of p-tau 181 are associated with postsynaptic pathology around Aβ plaques, while p-tau 181 signals are also associated with axonal abnormalities in the brain.

### p-tau 202/205/208 (AT8) did not colocalize with glial markers in the brains of *App^NLGF^* mice

**Figure 5:**
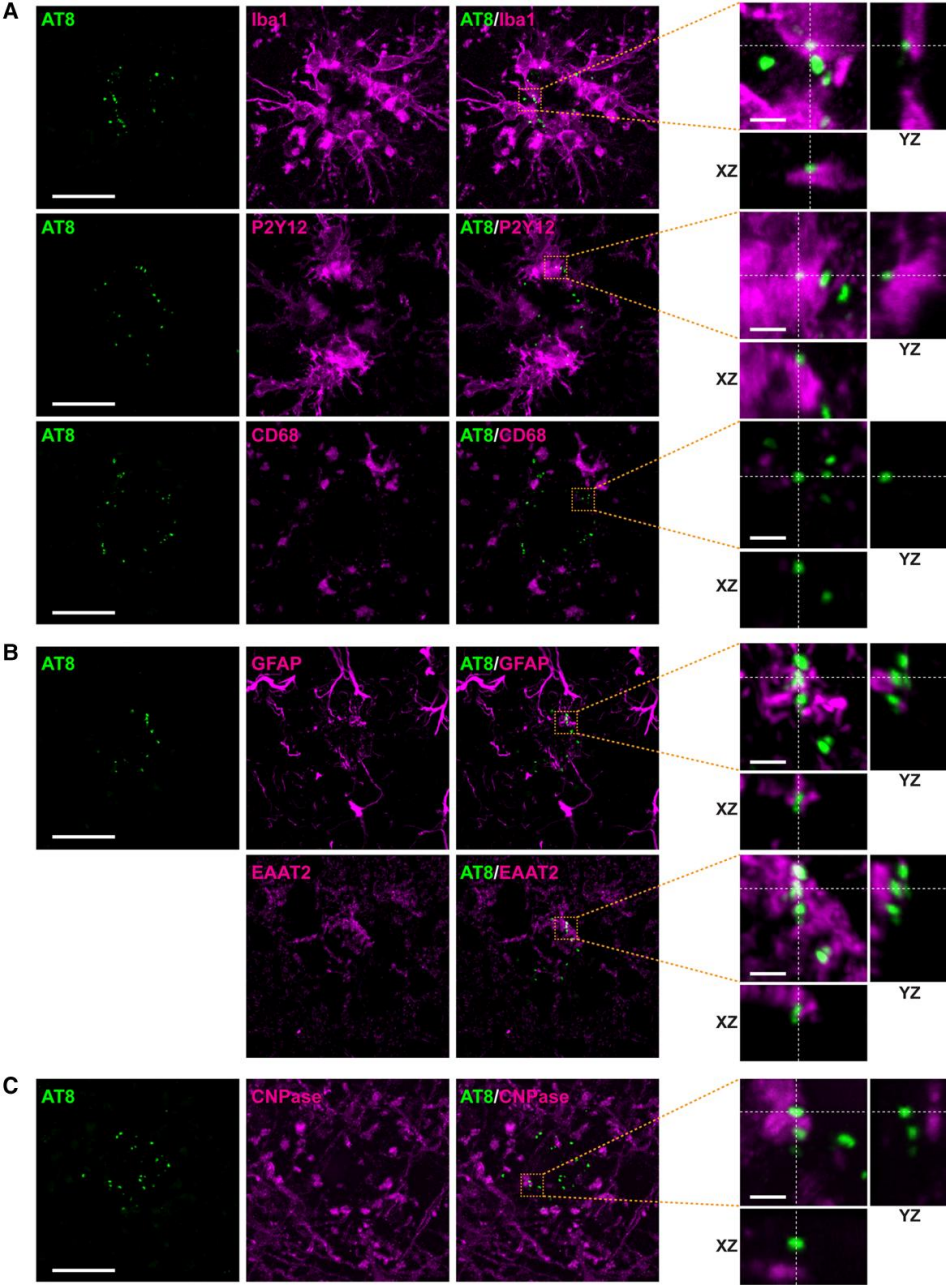
**Glial markers do not colocalize with p-tau 202/205/208 (AT8) in the brain of *App^NLGF^* mice.** Representative images of cortices from frozen coronal brain sections immunostained with (**A**) antibodies against p-tau 202/205/208 (AT8) and the microglial markers Iba1, P2Y12 and CD68 antibodies, (**B**) antibodies against AT8 and the astrocyte markers GFAP and EAAT2 and (**C**) antibodies against AT8 and the oligodendrocyte marker CNPase. The two AT8 images in **B** were analysed using sections co-stained with GFAP and EAAT2, with colocalizations examined in the same images as AT8 signals. Scale bars, 20 µm. Orthogonal views of different planes (*yz*, *xz*) of higher magnification images from the corresponding dashed orange squares are shown on the right; Scale bars in the orthogonal views, 2.5 µm.

Microgliosis has been observed in the brains of patients with Alzheimer’s disease, with recent studies showing that microglial cells around Aβ plaques engulf tau proteins and induce their propagation in mouse models.^56,57^ To determine whether punctate signals positive for p-tau 202/205/208 (AT8) around Aβ plaques colocalized with microglial cells, brain sections from *App^NLGF^* mice were stained with antibodies against p-tau 202/205/208 (AT8) and markers for the active microglial marker, ionized calcium-binding adapter molecule 1 (Iba1); the resting microglial marker, purinergic receptor P2Y12 (P2Y12) and the microglial lysosome marker, cluster of differentiation 68 (CD68). None of these microglial markers, however, colocalized with p-tau 202/205/208 (AT8) (Fig. 5A).

Astrocytes around Aβ plaques are also thought to be involved in phagocytosis.^58–62^ To determine whether p-tau 202/205/208 (AT8) signals colocalize with astrocytes, mouse brain sections were co-stained with antibodies against p-tau 202/205/208 (AT8) and the reactive astrocyte marker, glial fibrillary acidic protein (GFAP) or the resting astrocyte marker, excitatory amino acid transporter 2 (EAAT2). Neither of these astrocyte proteins, however, colocalized with p-tau 202/205/208 (AT8) (Fig. 5B). In addition, p-tau 202/205/208 (AT8) did not colocalize with the oligodendrocyte marker cyclic nucleotide phosphodiesterase (CNPase) (Fig. 5C). Taken together, these results suggest that p-tau- and PSD95-positive punctate structures around Aβ plaques are not likely engulfed by glial cells in the brains of *App^NLGF^* mice.

### Colocalization of p-tau 217, PSD95 and an active form of GSK3β in the brains of *App^NLGF^* mice

**Figure 6:**
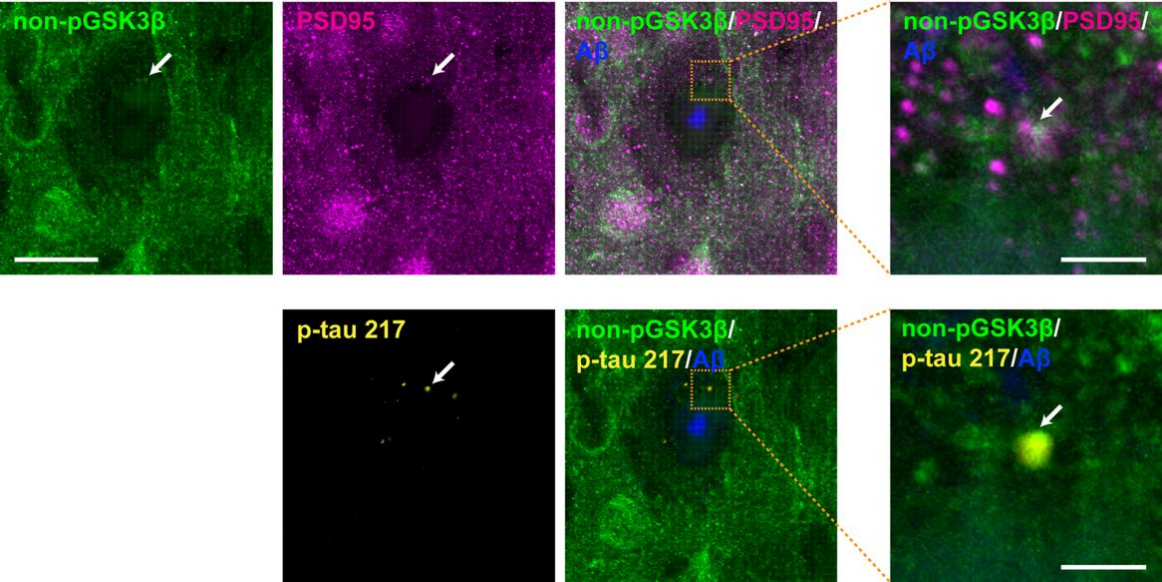
**Colocalization of p-tau 217, PSD95 and the active form of GSK3β in *App^NLGF^* mice.** Representative images of cortices from frozen coronal brain sections immunostained with antibodies against the active form of GSK3β (nonphospho-GSK3β) and PSD95 or p-tau 217. Aβ plaques are detected by staining with FSB. Scale bars, 20 µm. Higher magnification views of the corresponding dashed orange squares are shown in the right panel; scale bars, 2.5 µm. White arrows indicate colocalization.

Glycogen synthase kinase 3β (GSK3β) is a major kinase responsible for tau phosphorylation, including phosphorylation at Ser202, Thr205, Ser208, Thr217 and Thr231 residues.^63–65^ To determine whether GSK3β colocalizes with these p-tau molecules and PSD95, brain sections from *App^NLGF^* mice were stained with antibodies against the active form of GSK3β, p-tau 217 and PSD95. The results showed the colocalization of these three proteins (Fig. 6, white arrows and insets with orange squares), suggesting that tau phosphorylation by GSK3β could occur at postsynapses in the *App^NLGF^* mouse brain.

## Discussion

Tau proteins are hyperphosphorylated at multiple sites and aggregated into NFTs in Alzheimer’s disease brains.^66–70^ Among these tau proteins, p-tau 181, p-tau 217 and p-tau 231 in CSF and plasma are regarded as diagnostic biomarkers for Alzheimer’s disease.^6,32,71,72^ Immunohistochemical studies using brains obtained post-mortem from patients with Alzheimer’s disease have shown that p-tau 181, p-tau 217 and p-tau 231 are present in pretangles and matured NFTs in neurons and that the levels of these proteins correlate positively with Aβ burden,^25,26^ suggesting that these p-tau species may reflect early as well as late stages of NFT pathology in Alzheimer’s disease pathogenesis. Interestingly, however, increased levels of these p-tau proteins in CSF and plasma precede tau-PET positivity and well predict Aβ-PET positivity during the preclinical stage of Alzheimer’s disease.^28,29^ Moreover, studies in mouse models of familial Alzheimer’s disease and familial Danish dementia have shown that extracellular amyloidosis is sufficient to increase p-tau 181 and p-tau 217 levels in CSF in the absence of tau tangles.^73^ In this study, we demonstrated that p-tau 217, p-tau 231 and a fraction of p-tau 181 were detected as punctate structures around Aβ plaques only in *App^NLGF^* mice, but not in *App^NL^* or WT mice, suggesting that these p-tau molecules represent brain pathology induced by Aβ burden.

Our findings also suggest that p-tau 181 and p-tau 217 may represent distinct aspects of neuronal pathology caused by Aβ plaques in the brains. Recent studies have consistently reported that elevated levels of p-tau 181, p-tau 217 and p-tau 231 in CSF and plasma accurately differentiate Aβ-positive from Aβ-negative individuals among both cognitively unimpaired and cognitively impaired individuals.^16,24,28,44,74,75^ Several studies have also reported that p-tau 217 is slightly more sensitive than p-tau 181,^30,45,75^ suggesting that these p-tau species may reflect different aspects of brain pathologies. We found that p-tau 217 was specifically detected as punctate structures around Aβ plaques in the *App^NLGF^* mouse brains and only a part of these structures overlapped with p-tau 181. In contrast, p-tau 181 was readily detectable as fibre structures in WT and *App^NL^* mouse brains, whereas p-tau 181 signals formed enlarged aberrant fibres around the areas of Aβ plaques in the *App^NLGF^* mice. These results suggest that p-tau 217 and p-tau 181 may represent a distinct type of neuritic pathology around Aβ plaques. These quantitative and qualitative differences between p-tau 181 and p-tau 217 may affect their sensitivity in detecting the disease stages of Alzheimer’s disease.

Tau is a microtubule-binding protein that normally localizes to axons,^49^ and is also known to regulate synaptic plasticity in the postsynaptic compartment.^54,76^ Under pathological conditions, tau proteins are accumulated in the soma, dendrites and postsynapses of neurons.^49–55^ In addition, microglia around Aβ plaques have been shown to actively engulf and release phosphorylated-tau proteins, which may contribute to the propagation of tau in the brain.^56,57^ To identify the subcellular localization of p-tau signals around Aβ plaques, we performed immunohistochemical analyses with antibodies against various neuronal and glial marker proteins. This systematic analysis revealed that p-tau 217-, p-tau 231- and a part of p-tau 181-positive punctate structures around Aβ plaques colocalized with a postsynaptic marker PSD95, while p-tau 181-positive fibre structures overlapped with the axonal damage marker NfL. These results further support the hypothesis that p-tau 217 and p-tau 181 signals may represent a distinct type of neuronal pathology around Aβ plaques. Interestingly, recent reports have shown that increased plasma p-tau 217 is the most sensitive biomarker for predicting future cognitive decline at the preclinical stage,^77^ while increased plasma p-tau 181 is the most sensitive biomarker for predicting cognitive worsening at the prodromal stage.^31,43^

Tau proteins can be locally translated from mRNA transported to the postsynaptic region and this translation is regulated by neuronal activity at excitatory synapses stimulated by glutamate.^78,79^ In the context of Alzheimer’s disease, Aβ abnormally stimulates α-amino-3-hydroxy-5-methyl-4-isoxazolepropionic acid and *N*-methyl-D-aspartic acid receptors at postsynapses and activates kinases such as Fyn kinase and GSK3β,^52,80^ which can phosphorylate tau proteins at Alzheimer’s disease related sites.^64,65^ These reports suggest that p-tau proteins could be phosphorylated in the postsynaptic region of the Alzheimer’s disease brains. Supporting this, we found that p-tau 217 colocalized with activated GSK3β along with a postsynaptic marker, PSD95, suggesting that Aβ pathology may cause aberrant postsynaptic activity, which induces local translation of tau and subsequent tau phosphorylation at postsynaptic sites. Increased levels of p-tau 217, p-tau 231 and p-tau 181 in CSF and plasma may therefore reflect aberrant activity in the postsynapses surrounding Aβ plaques, which may be related to the neuronal hyperexcitability observed in the early stage of Alzheimer’s disease.^81,82^

Tau proteins are truncated and secreted by neurons into the extracellular space and most are detected in the CSF as *N*-terminal fragments.^44,83,84^ The results of the present study showed that p-tau 217-, p-tau 231- and some p-tau 181-positive signals localized around Aβ plaques, although it was unclear whether these p-tau species were full-length or truncated forms of tau. Moreover, the mechanisms by which these p-tau proteins are secreted into the CSF and plasma remain unknown. Elucidation of these mechanisms may enable the utilization of soluble biomarkers to evaluate potential treatments for Alzheimer’s disease.

Six tau isoforms with three microtubule-binding domain repeats (3R tau) or four microtubule-binding repeats (4R tau) are expressed in human brain and aggregated into tau pathologies in patients with Alzheimer’s disease.^85^ One of the limitations of this study using mouse models is that only three 4R tau isoforms are expressed in the mouse brain.^51,86^ Although the primary structures of human and mouse tau proteins are highly conserved, they differ in several amino acids, especially in their *N*-terminal regions. These differences may influence their ability to aggregate, their phosphorylation profile and their metabolism. Additional studies using human tau and *App^NLGF^* double knock-in mice, which express all six isoforms of human tau with Aβ amyloidosis,^84^ may address these issues.

Most importantly, key findings from mouse models must be validated in human brains. A recent study using post-mortem Alzheimer’s disease brains demonstrated that p-tau 217 was detected in NFTs and neuropil threads, which are also positive for p-tau 181, 202, 202/205, 231 and 369/404.^25^ Interestingly, p-tau 217 was colocalized with granulovacuolar degeneration bodies and multi-vesicular bodies markers.^25^ However, since increases in the level of p-tau 217, p-tau 231 and p-tau 181 in CSF and plasma are well correlated with Aβ plaques before NFTs formation and the brains used in the study were from the advanced stage of Alzheimer’s disease,^25^ it will be important to analyse p-tau signals in the brains at the preclinical stage of Alzheimer’s disease.

## Conclusion

This study demonstrated that the CSF and plasma Alzheimer’s disease biomarkers p-tau 181 and p-tau 217 represent a distinct type of neuritic pathology around Aβ plaques in *App* knock-in mouse models of Aβ amyloidosis. The presence of p-tau 217, p-tau 231 and a fraction of p-tau 181 reflects postsynaptic pathology, with p-tau 181 also representing axonal abnormality around Aβ plaques. These results suggest that proteins specifically associated with neuritic pathology around Aβ plaques could have potential as CSF and plasma biomarkers of the preclinical stage of Alzheimer’s disease and that mouse models of Aβ amyloidosis may be useful for identifying such biomarkers.

## Supplementary Material

fcac286_Supplementary_Data

## Abbreviations

Aβ =: amyloid-β
BSA =: bovine serum albumin
CD68 = =: cluster of differentiation 68
CNPase =: cyclic nucleotide phosphodiesterase
CSF =: cerebrospinal fluid
EAAT2 =: excitatory amino acid transporter 2
FSB =: 1-fluoro-2,5-bis (3-carboxy-4-hydroxystyryl) benzene
GFAP =: glial fibrillary acidic protein
GSK3β =: glycogen synthase kinase 3β
5-HT = =: 5-hydroxytryptamine
Iba1 =: ionized calcium-binding adapter molecule 1
LAMP1 =: lysosomal-associated membrane protein 1
LC =: locus coeruleus
MAP2 =: microtubule-associated protein 2
NfL =: neurofilament light chain
NFTs =: neurofibrillary tangles
P2Y12 =: purinergic receptor P2Y12
PBS =: phosphate-buffered saline
PBS-T =: PBS containing 0.1% Triton X-100
PET =: Positron emission tomography
PSD95 =: postsynaptic density protein 95
p-tau 181 =: tau phosphorylated at Thr181
p-tau 217 =: tau phosphorylated at Thr217
p-tau 231 =: tau phosphorylated at Thr231
VAChT =: vesicular acetylcholine transporter
VGAT =: vesicular GABA transporter
VGLUT1 =: vesicular glutamate transporter 1
WT =: wild-type
