# Supplementary material for: Distinct brain pathologies associated with Alzheimer’s disease biomarker-related phospho-tau 181 and phospho-tau 217 in App knock-in mouse models of amyloid-β amyloidosis
Source: Brain Commun. 2022 Nov 6;4(6):fcac286. doi: 10.1093/braincomms/fcac286 (PMC9683396; doi:10.1093/braincomms/fcac286)
Supplement: fcac286_Supplementary_Data [file fcac286_supplementary_data.pdf]

Supplementary data

Distinct brain pathologies associated with Alzheimer's disease biomarker-related phospho-tau 181 and phospho-tau 217 in *App* knock-in mouse models of Aβ amyloidosis

Yu Hirota[1, 2], Yasufumi Sakakibara[1], Kyoko Ibaraki[1], Kimi Takei[1], Koichi M. Iijima[1, 3, *], Michiko Sekiya[1, 3, *]

[1]Department of Neurogenetics, Center for Development of Advanced Medicine for Dementia, National Center for Geriatrics and Gerontology, Obu, Aichi, Japan 474-8511

[2]Research Fellow of Japan Society for the Promotion of Science, Chiyoda-ku, Tokyo, Japan 102-0083

[3]Department of Experimental Gerontology, Graduate School of Pharmaceutical Sciences, Nagoya City University, Nagoya, Japan 467-8603

*Corresponding authors

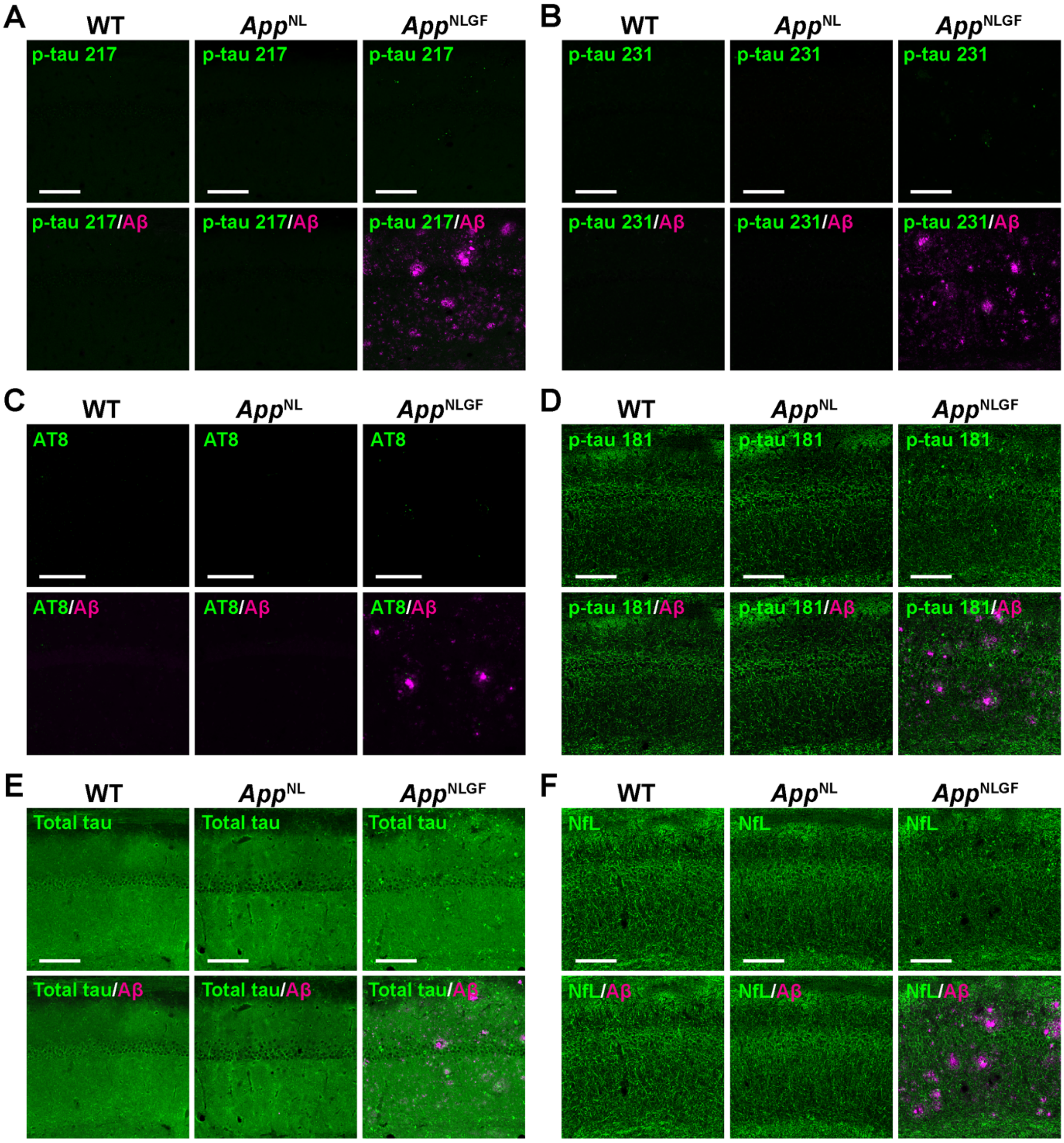

Supplementary Figure 1. Presence of p-tau 217, 231, 202/205/208 (AT8) and 181 around Aβ plaques in CA1.

Representative images of the CA1 region of the hippocampus from frozen coronal brain sections immunostained with antibodies against (**A**) p-tau 217, (**B**) p-tau 231, (**C**) p-tau 202/205/208 (AT8), (**D**) p-tau 181, (**E**) total tau, and (**F**) NfL (each in green) in combination with an antibody against Aβ (in magenta). Scale bars, 100 μm.

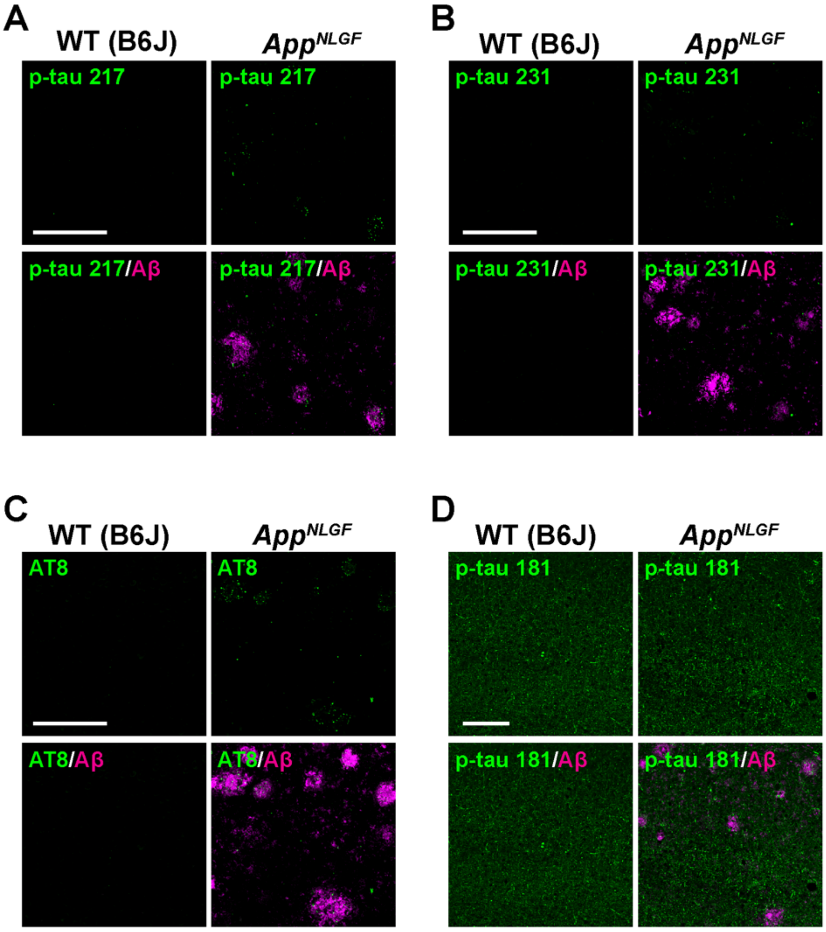

Supplementary Figure 2. Presence of p-tau 217, 231, 202/205/208 (AT8) and 181 around Aβ plaques in ***App***^{NLGF} **female mice.**

Representative images of cortices from frozen coronal brain sections of female mice immunostained with antibodies against (**A**) p-tau 217, (**B**) p-tau 231, (**C**) p-tau 202/205/208 (AT8) and (**D**) p-tau 181 (each indicated by green) in combination with an antibody against Aβ (magenta). Scale bars, 100 µm.

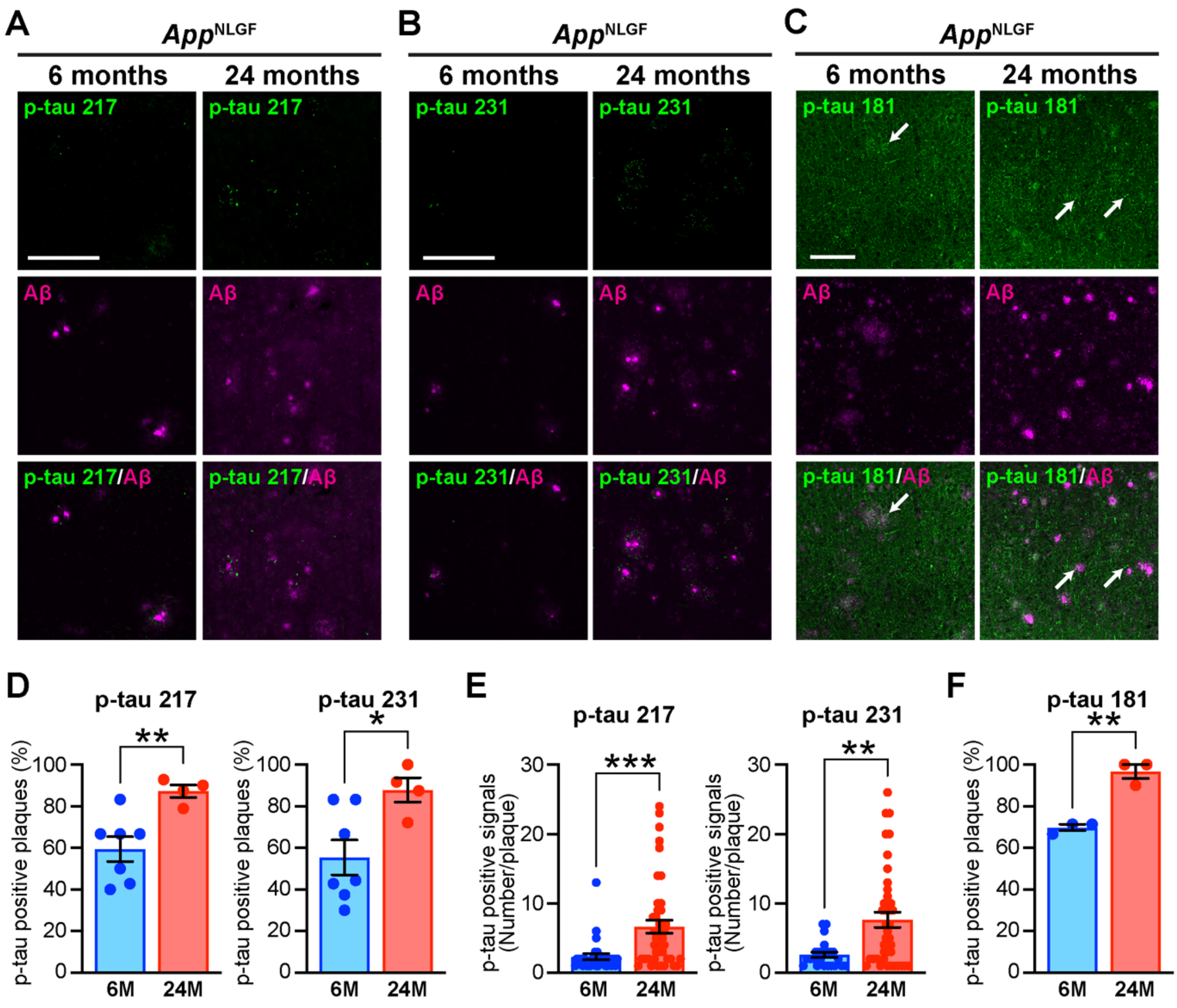

Supplementary Figure 3. Age-associated increases in the number of p-tau 217 and 231 signals around Aβ plaques.

Representative images of the cortices from brain sections of 6- and 24-month-old *App*^{NLGF} mice immunostained with antibodies against (**A**) p-tau 217, (**B**) p-tau 231, and (**C**) p-tau 181 (each in green). Aβ plaques are detected by staining with FSB or Aβ antibody (in magenta). Scale bars, 100 μm. (**D-F**) Quantification analyses for the ratio of Aβ plaques with or without p-tau signals and the number of p-tau signals around Aβ plaques in both 6- and 24-month-old *App*^{NLGF} mice. Mean ± SEM. n=4-7 sections (p-tau 217 and 231), n=3 sections (p-tau 181), $^{*}p<0.05$, $^{**}p<0.01$ and $^{***}p<0.001$ vs. 6-month-old by unpaired Student's *t*-test.

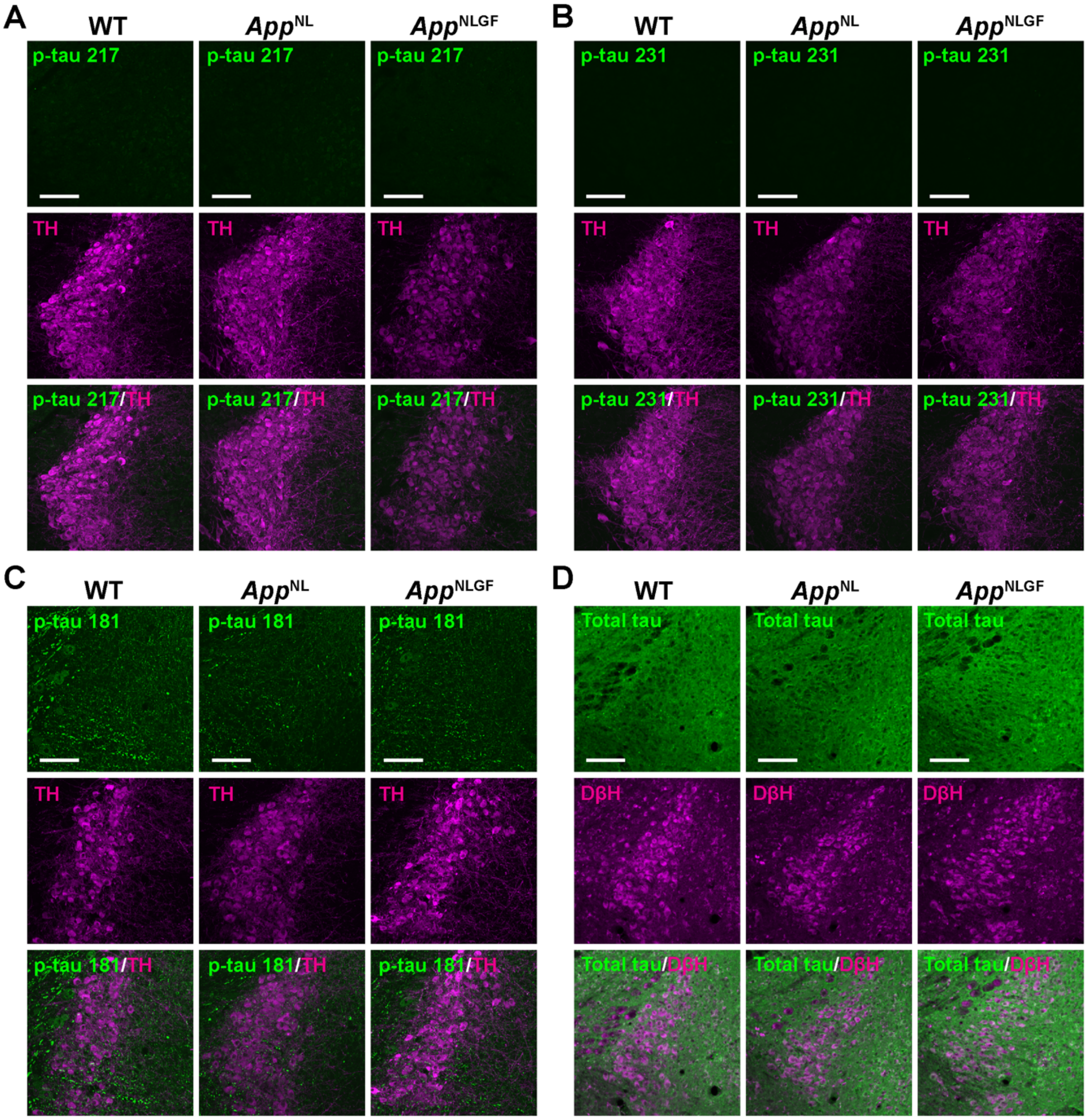

Supplementary Figure 4. Absence of p-tau 217 and 231 from the LC of *App*^{NLGF} mouse brain. Representative images of the locus coeruleus (LC) from frozen coronal brain sections immunostained with antibodies against (**A**) p-tau 217, (**B**) p-tau 231, (**C**) p-tau 181, and (**D**) total tau (each in green) and with antibodies against the noradrenergic neuronal markers, tyrosine hydroxylase (TH) and dopamine β-hydroxylase (DβH) (each in magenta). Scale bars, 100 µm.

Supplementary Table 1. Antibodies used in this study.

REAGENT or RESOURCE	SOURCE	IDENTIFIER	RRID	DILUTION
Primary antibodies				
Mouse monoclonal anti-Aβ-N [82E1]	IBL	10323	AB_10707424	1:200
Rabbit polyclonal anti-Aβ-N	IBL	18584	AB_10705431	1:200
Mouse monoclonal anti-p-tau 202/205/208 [AT8]	Thermo Fisher	MN1020	AB_223647	1:500
Rabbit monoclonal anti-p-tau 202/205 [AH36]	StressMarq	SMC-601D	AB_2820300	1:500
Mouse monoclonal anti-p-tau 181 [AT270]	Thermo Fisher	MN1050	AB_223651	1:500
Rabbit polyclonal anti-p-tau 217	Thermo Fisher	44-744	AB_2533741	1:500
Mouse monoclonal anti-p-tau 231 [AT180]	Thermo Fisher	MN1040	AB_223649	1:500
Rat monoclonal anti-tau [RTM38]	Fujifilm Wako Chemicals	017-26893	N/A	1:300
Rabbit monoclonal anti-Neurofilament-L [C28E10]	Cell Signaling	2837S	AB_823575	1:300
Rabbit polyclonal anti-MAP2	Millipore	AB5622	AB_91939	1:200
Rabbit polyclonal anti-Bassoon	Synaptic Systems	141-002	AB_887698	1:1000
Guinea pig polyclonal anti-PSD95	Frontier Institute	PSD95-GP-Af660	AB_2571539	1:300
Mouse monoclonal anti-nonphospho-GSK3β (S9) [12B2]	Millipore	MABN2443	AB_2832942	1:200
Rabbit polyclonal anti-vesicular glutamate transporter 1 (VGLUT1)	Synaptic Systems	135-303	AB_887875	1:1000
Rabbit polyclonal anti-vesicular GABA transporter (VGAT)	Millipore	AB5062P	AB_2301998	1:500
Rabbit polyclonal anti-vesicular acetylcholine transporter (VAChT)	Frontier Institute	VAChT-Rb-Af1000	AB_2571850	1:300
Rabbit polyclonal anti-5-HT	Immunostar	20080	AB_572263	1:500
Chicken polyclonal anti-tyrosine hydroxylase (TH)	Abcam	ab76442	AB_1524535	1:1500
Rabbit polyclonal anti-dopamine β hydroxylase (DβH)	Immunostar	22806	AB_572229	1:500
Rabbit polyclonal anti-Iba1	Fujifilm Wako Chemicals	019-19741	AB_839504	1:500
Rat monoclonal anti-CD68 [FA-11]	Bio-Rad Laboratories	MCA1957	AB_322219	1:200
Rabbit polyclonal anti-P2Y12	AnaSpec	55043A	AB_2298886	1:500
Rat monoclonal anti-GFAP [2.2B10]	Millipore	345860	AB_2109651	1:1000
Rabbit polyclonal anti-EAAT2	Abcam	ab41621	AB_941782	1:300
Rabbit monoclonal anti-CNPase [D83E10]	Cell Signaling	5664S	AB_10705455	1:100

Secondary antibodies				
Goat polyclonal anti-Mouse IgG H&L Alexa Fluor 488	Abcam	ab150117	AB_2688012	1:500
Goat polyclonal anti-Mouse IgG H&L Alexa Fluor 594	Abcam	ab150120	AB_2631447	1:500
Goat polyclonal anti-Mouse IgG H&L Alexa Fluor 647	Abcam	ab150119	AB_2811129	1:500
Goat polyclonal anti-Rabbit IgG H&L Alexa Fluor 488	Abcam	ab150081	AB_2734747	1:500
Goat polyclonal anti-Rabbit IgG H&L Alexa Fluor 594	Abcam	ab150084	AB_2734147	1:500
Goat polyclonal anti-Rabbit IgG H&L Alexa Fluor 647	Abcam	ab150083	AB_2714032	1:500
Goat polyclonal anti-Rat IgG H&L Alexa Fluor 594	Abcam	ab150168	N/A	1:500
Goat polyclonal anti-Rat IgG H&L Alexa Fluor 647	Abcam	ab150167	AB_2864291	1:500
Goat polyclonal anti-Guinea Pig IgG H&L Alexa Fluor 594	Thermo Fisher	A-11076	AB_2534120	1:500
Goat polyclonal anti-Chicken IgY H&L Alexa Fluor 594	Abcam	ab150176	AB_2716250	1:500
Counterstain				
DAPI (4',6-Diamidino-2-Phenylindole, Dihydrochloride)	Thermo Fisher	D1306	AB_2629482	5.7 µM
FSB (1-Fluoro-2,5-bis(3-carboxy-4-hydroxystyryl)benzene, DMSO solution)	Dojindo	F308	N/A	23.8 µM
